# Innovative BIM technology application in the construction management of highway

**DOI:** 10.1038/s41598-024-66232-5

**Published:** 2024-07-03

**Authors:** Dong Zhou, Bida Pei, Xueqin Li, Ding Jiang, Lin Wen

**Affiliations:** 1 Changsha University of Science and Technology, Changsha, China; 2 Changsha BIM Cloud Information Technology Co., Ltd, Changsha, China; 3 Hunan Expressway Group Co., Ltd, Changsha, China; 4 JiangXi Ganyue Expressway Engineering Co., Ltd, Nanchang, China

**Keywords:** Building Information Modeling (BIM), Highway, Construction Management, Construction Simulation, Geographic Information Systems (GIS), Civil engineering, Computational science

## Abstract

Within the global architecture, engineering, and construction industry, the use of Building Information Modeling (BIM) technology has significantly expanded. However, given the unique characteristics of road infrastructure, the application of BIM technology is still being explored. This article focuses on the Yuanchen Expressway, exploring innovative applications of BIM technology in comprehensive construction management. The project employs advanced technologies, including BIM, Geographic Information Systems (GIS), and the Internet of Things (IoT), to precisely identify critical nodes and breakthroughs. Supported by a detailed BIM model and a multi-level, diversified digital management platform, the project effectively addresses construction challenges in multiple tunnels, bridges, and complex interchanges, achieving intelligent construction innovation throughout the Yuanchen Expressway with BIM technology. By guiding construction through BIM models, utilizing a BIM+GIS-based management cloud platform system, and employing VR safety briefings, the project effectively reduces the difficulty of communication and coordination in project management, shortens the project measurement cycle, improves on-site work efficiency, and ensures comprehensive control and safety management. This article provides an exemplary case for the application of full-line construction management using BIM technology in the highway sector both in China and globally, offering new perspectives and strategies for highway construction management.

## Introduction

Since 2015, the Chinese government has consistently underscored the advancement and integration of BIM technology, incorporating it into pivotal strategies for industrialized housing and progressive developments in the construction sector. Within the transportation domain, China’s Ministry of Transport actively champions the digital transformation of roads and the establishment of intelligent highways. These policies incentivize construction entities to synchronize planning efforts, formulate comprehensive life cycle models, engage diverse stakeholders, and digitalize engineering management to attain cost reduction and efficiency enhancement. Commencing June 2024, newly initiated national highway projects are mandated to submit BIM design results, while other projects are strongly encouraged to embrace BIM design technology^1^. Looking ahead, a digitalized highway network is poised to become an integral element of emerging development paradigms.

### Historical Development of BIM technology

BIM technology, represents a digitized approach to architectural design, construction, and operation grounded in digital technology. It streamlines the integration and management of information across the entire lifecycle of a construction project^2^. The theoretical roots of BIM technology trace back to the 1970s in computer-aided design and the architectural domain. With enhanced computing capabilities and the evolution of 3D modeling technology, BIM has gradually surfaced. Its evolutionary stages encompass:

- *Basic modeling* In its initial phases, BIM technology predominantly involved 3D modeling for architectural design, replacing traditional 2D drawings.
- *Multidimensional data integration* Advancing BIM technology incorporated additional dimensions such as time, cost, and materials to bolster construction and management processes.
- *Cloud computing and mobile applications* In recent years, BIM technology has migrated to the cloud, fostering more convenient collaboration and supporting the development of mobile applications.
- *Smart buildings and the IoT* Presently, the fusion of BIM technology with the IoT propels smart building and equipment management, enhancing building efficiency and sustainability.

The application of BIM in architecture spans project stages, aiding feasibility analysis, simulation, optimization, and efficiency assessments for designers^3–6^. Construction teams use BIM for detailed design, simulation, schedule management, and resource scheduling, enhancing quality and efficiency^7,8^.Operation teams rely on BIM for equipment management, energy monitoring, and maintenance, ensuring long-term reliability^9–12^. BIM improves efficiency, quality, and collaboration, fostering transparency, traceability, customer satisfaction, and social responsibility.

### BIM technology in road infrastructure

Highway construction confronts considerable challenges, demanding more efficient and precise construction management methods to meet requirements and uphold project quality. AngYang emphasizes the need to intensify BIM application in road infrastructure^13^. Instances of BIM technology applications in road infrastructure abound, with notable cases including:

- *Hong Kong–Zhuhai–Macao Bridge (HZMB)* Spanning 55 kilometers with bridges, tunnels, and artificial islands, employed BIM for design, construction, and maintenance. This enhanced collaboration, ensuring project quality, safety, and cost savings^14^.
- *Beijing daxing International Airport* The world’s largest single-terminal airport, utilized BIM for planning, design, construction supervision, and maintenance. This integration boosted project efficiency and overall benefits^15^.
- *Shenzhen metro* One of China’s largest metro projects, spanning 303 kilometers across 11 lines, prominently utilized BIM for simulation analysis and optimization in underground planning, route design, station layout, and tunnel construction. This proactive strategy effectively addressed challenges tied to complex geological conditions, significantly enhancing metro construction safety and reliability^16^.

In the realm of road infrastructure, the application of BIM technology primarily emphasizes the following aspects:

- *BIM in bridge engineering* BIM technology in bridge engineering improves design quality, construction efficiency, progress control, and ongoing structural monitoring. ShenJian and team explored bridge construction safety management with BIM, leading to a proposed BIM-centric system for enhanced safety measures^17^. Marzouk and Hisham delved into the application of BIM in cost and time management within bridge engineering, culminating in the development of a BIM-based bridge lifecycle cost model^18^.
- *BIM in tunnel engineering* BIM technology enhances tunnels by visualizing geological conditions, optimizing design, simulating construction, and managing maintenance. Cudrigh-Maislinger’s BIM pilot project, the Karawanken Tunnel, exemplifies BIM’s potential in overseeing complex infrastructure projects like tunnel construction^19^. In the ambitious nearly 7-kilometer tunnel boring machine project in Berlin’s city center, Fentzloff amalgamated BIM with Lean principles, illustrating how the combined approach enhances tunnel construction efficiency, safety, and quality by minimizing waste, enhancing communication, and expediting decision-making^20^.
- *BIM in road engineering *Highways utilize BIM for efficient route planning, pavement design, construction, and ongoing monitoring. Researchers, including Vignali and Valeria, applied BIM to upgrade existing road infrastructure, creating 3D models for both old and new elements. Simulations and analyses for traffic, safety, environment, and economics led to optimized designs, reduced construction time and costs, and improved maintenance and management of road infrastructure^21^. Kohlböc demonstrated BIM’s application in building a 21-kilometer rail segment from Köstendorf to Salzburg, encompassing tunnels, bridges, and viaducts. The project utilized a digital parametric foundation model, integrating geological and geotechnical characteristics, for comprehensive design precision^22^. Kim and colleagues applied BIM technology to a highway project, developing an object-based intelligent model capable of automatically obtaining cost and schedule estimates within the model^23^.

The widespread integration of BIM technology in the realm of road infrastructure yields myriad advantages for engineering projects, augmenting efficiency, quality, and contributing to the sustainable construction of road infrastructure. However, the implementation of BIM in the road infrastructure sector still faces many challenges.

- BIM was initially developed for vertical buildings; however, infrastructure such as roads, railways, airport runways, dams, and bridges possess linear characteristics and extensibility, making it challenging to model them using the same schemes and principles as building BIM^24^. To address this issue, Barazzetti utilized GIS technology to provide precise geospatial data and analytical capabilities, supporting the efficient modeling and management of road infrastructure^25^.
- The effective implementation of BIM, as an emerging technology, requires support from professional knowledge and skills. However, a lack of comprehensive BIM talent cultivation systems both domestically and internationally results in insufficient understanding and knowledge among participants, limiting the full potential of BIM^26,27^.
- The involvement of multiple stakeholders in road infrastructure projects highlights the necessity for an effective communication and collaboration platform. Unfortunately, a unified BIM platform is lacking both domestically and internationally. Diverse stakeholders using different software and tools result in issues like information isolation, data redundancy, and version confusion, impacting collaborative management effectiveness^28^.

Despite the challenges, BIM technology is expected to continue playing a crucial role in the fields of building and road infrastructure, supporting more efficient and sustainable engineering management and construction. It offers new tools and methods for innovative applications in the comprehensive construction management of highways.

## Project overview

**Figure 1:**
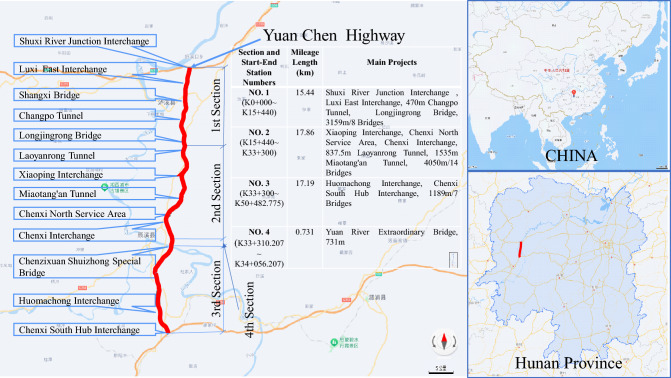
Yuan Chen highway. (This map was generated using Baidu Maps (version 20.1.0, URL: https://map.baidu.com/search/%E6%B2%85%E8%BE%B0%E9%AB%98%E9%80%9F/@12273808.185,3242640.65,11.1z?querytype=s &da_src=shareurl &wd=%E6%B2%85%E8%BE%B0%E9%AB%98%E9%80%9F &c=158 &src=0 &pn=0 &sug=0 &l=13 &b=(12523651,2936176;12583555,2965744) &from=webmap &biz_forward=%7B%22scaler%22:1,%22styles%22:%22pl%22%7D &device_ratio=1), and then we further processed it using PowerPoint.).

The Yuan Chen Highway (Figure 1) stands as a significant road infrastructure endeavor situated in China. Commencing at Shuxi Kou in Pangu Township, Yuanling County, Hunan Province, it interconnects with the Changji Highway. The Highway traverses Changpo, Tianwan, the eastern sector of Chenxi County, Shibei, and culminates at Muqiaojiang in Huomachong Town, linking seamlessly with the Xuhuai Highway. The project is segmented into four distinct construction bid packages, with an anticipated total investment of 7.036 billion yuan. The mainline spans approximately 50.483 kilometers, and the construction timeline is set at 36 months, featuring a roadbed width of 26 meters. Designed as a dual two-lane configuration, the Highway boasts a designated design speed of 100 kilometers per hour.

### Major projects

The Yuan Chen Highway encompasses several pivotal engineering components, including interchanges, tunnels, bridges, and service areas. These projects present specific technical challenges that necessitate innovative solutions. The distinct engineering components are detailed as follows:

- *Interchange engineering* The Highway boasts a total of six interchanges. Notably, the Chenxi Interchange adopts a single-column elevated bridge structure, featuring a mainline bridge length of 120 meters and a ramp bridge length of 60 meters.
- *Tunnel engineering* With a total of three tunnels, the Highway’s most extended tunnel is the Miaotang’an Tunnel, spanning 1535 meters. The construction methodology adheres to arched advance principles and incorporates integral lining of the arch wall. This process utilizes a full-section hydraulic formwork for step-by-step pouring.
- *Bridge engineering* The Highway incorporates six bridges, with particular emphasis on the Yuan River Extra-Large Bridge as a critical control project. Linking Yuanling and Chenxi, this bridge spans the Yuan River, with a total length of 732 meters. It employs a continuous rigid frame bridge type, featuring separate lanes on the left and right.
- *Service area engineering* Singularly, the Highway accommodates one service area-the Chenxi North Service Area. Situated in Huomachong Village, Huomachong Town, Chenxi County, it stands as one of the inaugural main building projects of the Yuan Chen Highway.

### Project characteristics

The project faces complex geological challenges, including subsidence areas, posing safety control difficulties. Segments from K32+050 to K33+500 show subsidence due to refractory clay mining, and areas from K12+700 to K13+200 reveal private coal mining. Among the 40 bridges, 24 are in karst or intense subsidence regions, requiring stringent embankment and bridge foundation treatments. Changpo Tunnel’s rock is mostly Grade V limestone, presenting challenges for construction safety.

The project intersects with the existing Highway at multiple points, heightening safety risks during construction. Spanning six bridges across national and provincial trunk road S250, the Yuan Chen Highway faces complex road conditions and heavy traffic flow. Six interchanges, including two connecting to existing Highways, add intricacy and coordination challenges, elevating safety risks.

The Yuan River Extra-Large Bridge, a control project, encounters challenges due to environmental constraints. Limited by the Yuan River channel, large lifting equipment and construction vessels are impractical. Consequently, the construction of pier 5’s steel cofferdam requires a “crawler crane modular assembly + jacking down” method, intensifying on-site construction challenges and precision control of cofferdam jacking.

## Application of BIM technology

Within this project, a sophisticated BIM model was crafted through the integration of technologies like GIS and the IoT. This integration facilitated the creation of a multi-level, multi-dimensional digital management platform. This optimization significantly improved project process management and decision support systems, showcasing innovative implementation for the intelligent construction of the entire Yuanchen Highway through the utilization of BIM technology.

### Establishment of BIM model

**Figure 2:**
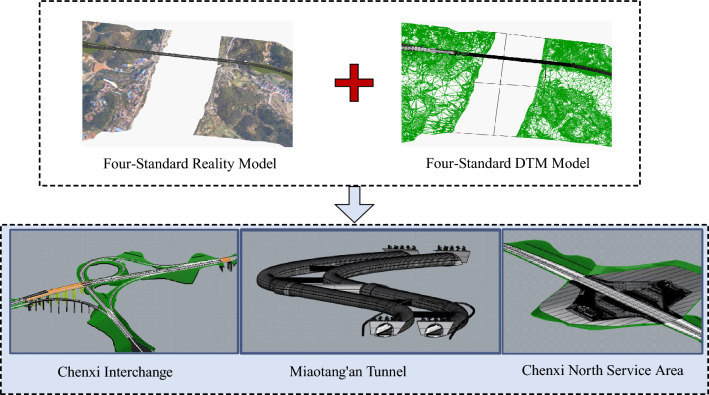
Establishing BIM model.

To construct a comprehensive BIM model, advanced technologies, including 5G and drone technology, were harnessed in conjunction with conventional oblique photogrammetry techniques. The creation of an accurate three-dimensional reality model was facilitated through the application of Context Capture. Subsequently, Pointools and Descartes were employed to derive Digital Terrain Models (DTM) from map data. Interchange design utilized OpenBridge Modeler, tunnel modeling was executed using Rhino, and road and service area modeling were conducted with MicroStation and OpenRoads Designer. Lastly, Aglosgeo played a crucial role in establishing a geological model, harmonizing and aligning 3D models from diverse sources and types. This accomplished a seamless integration of 3D geological features, terrain, and real-world conditions, resulting in the development of a lifelike construction environment. The detailed process is elucidated in (Figure 2).

### BIM detailed design

#### Parametric model establishment

The reinforcement modeling process in the project, involving considerations like shape, dimensions, and concrete beam sections, is complex and time-consuming. Details such as diameter, spacing, and connections are crucial. To enhance efficiency, a rapid reinforcement modeling plugin was developed using Grasshopper and Rhino software. This innovative tool simplifies the modeling process, providing an efficient and precise solution for reinforced structural design.

**Figure 3:**
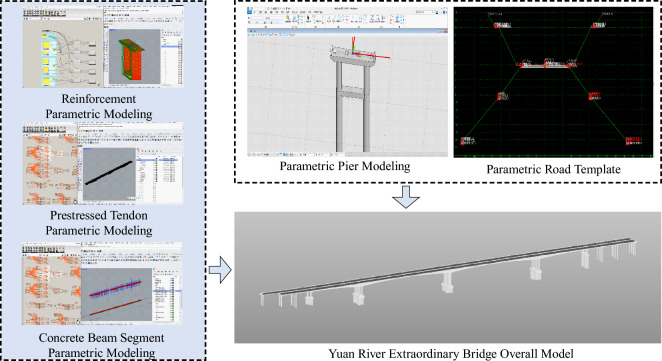
Parametric model.

Expanding the plugin’s application to MicroStation, a parametric section template for the bridge was created. Simultaneously, through OpenBridge Modeler, we achieved the parametric establishment of the bridge pier model. The integration of these five models resulted in the comprehensive modeling of the entire bridge main structure, as illustrated in (Figure 3). This innovative approach not only increased work efficiency but also ensured modeling accuracy and consistency.

#### Clash detection

Clash detection, facilitated by BIM technology, is a spatial analysis method for three-dimensional models that automatically identifies geometric clashes within locally sourced content. This process allows for the detection and resolution of conflicts and interferences in the design phase, thereby mitigating construction costs and risks, and enhancing engineering quality and efficiency.

**Figure 4:**
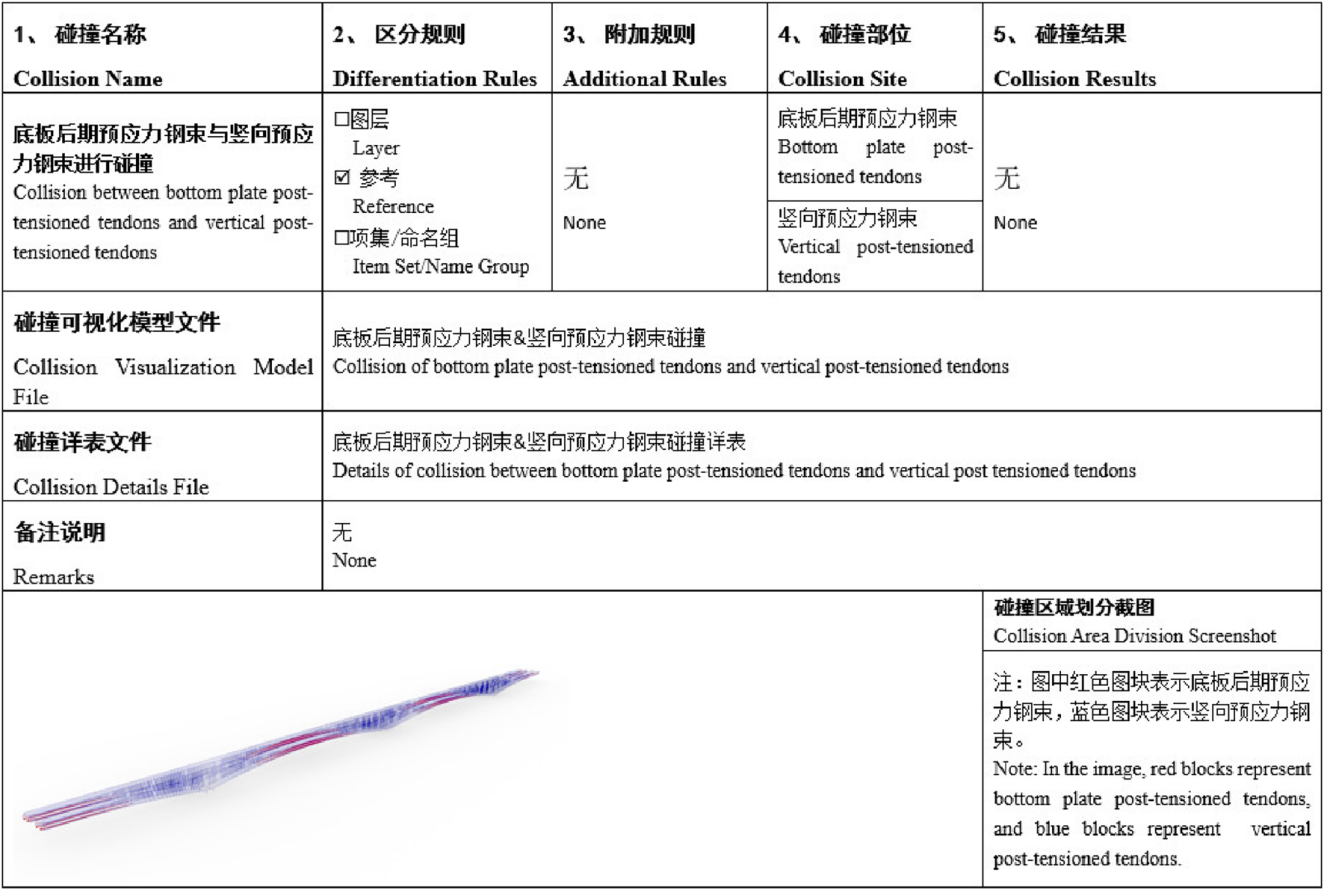
Clash detection report.

In this project, Bentley software was employed for clash detection, successfully identifying and resolving more than 50 clash issues (Figure 4), encompassing both hard and soft clashes. Timely grouping, marking, annotation, and resolution of clash issues were meticulously executed. The results were compiled into a comprehensive clash report, which underwent communication and negotiation with various discipline designers. The clash detection process played a pivotal role in ensuring the safety and reliability of node locations, optimizing the design, and ultimately improving construction efficiency.

### Intelligent application of BIM models

#### Simulation of construction organization for key projects

Given the intricate nature of the Yuanchen Highway, we employed construction organization simulation animation to replicate key construction processes along the entire route. This animation, leveraging BIM technology, integrates the three-dimensional model of a structure with construction scheduling, presenting the construction process in an animated format. Its objectives encompass:

- Early identification and resolution of issues in design and construction, optimization of construction plans, cost savings, acceleration of construction progress, and control of construction quality.
- Clear demonstration of construction challenges, enhancing operational efficiency, and fostering safety awareness among construction personnel.
- Establishment of effective coordination and management among various construction disciplines, mitigating conflicts and errors.
- Facilitation of communication and collaboration, providing stakeholders, supervisors, and designers with a more intuitive understanding of project details.

To accomplish construction organization simulation animation, several steps are essential: first, create the project’s three-dimensional model using Bentley software, add relevant attribute information, and export the 3D model as FBX or DWG files. Next, utilize 3DMax to edit and render the 3D model, add animation effects, and finally, employ Adobe After Effects to edit, composite, color-correct, and add special effects to the video, resulting in the construction organization simulation animation.

**Figure 5:**
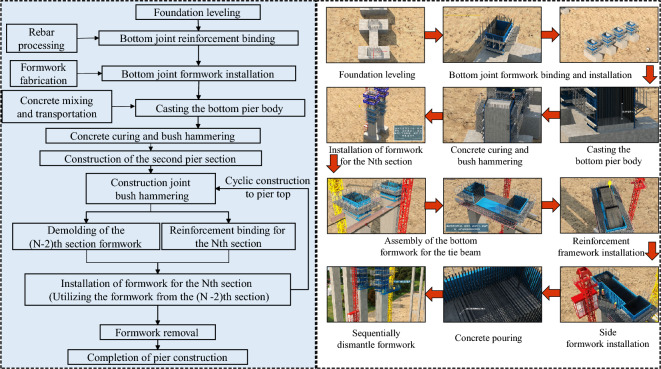
Specialized construction simulation of bridge high-pier columns.

**Figure 6:**
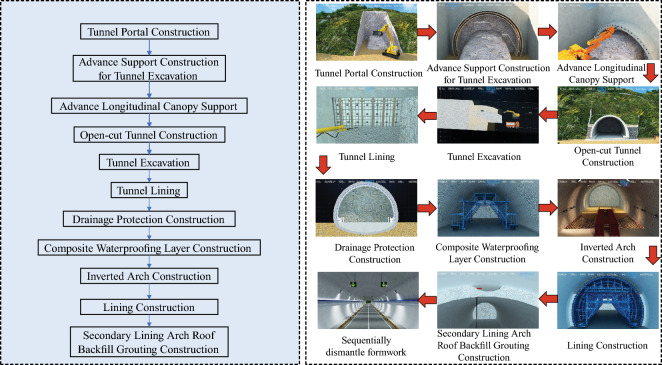
Tunnel construction simulation.

**Figure 7:**
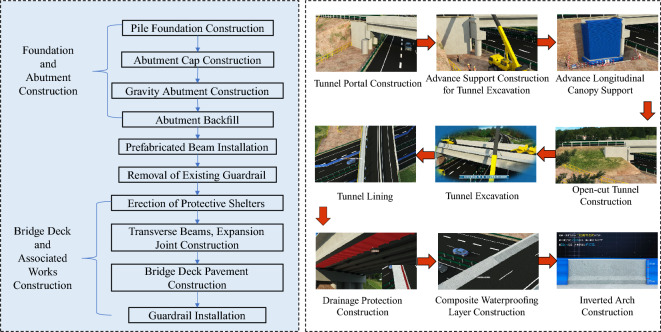
Construction simulation of Chenxi South hub interchange bridge widening.

**Figure 8:**
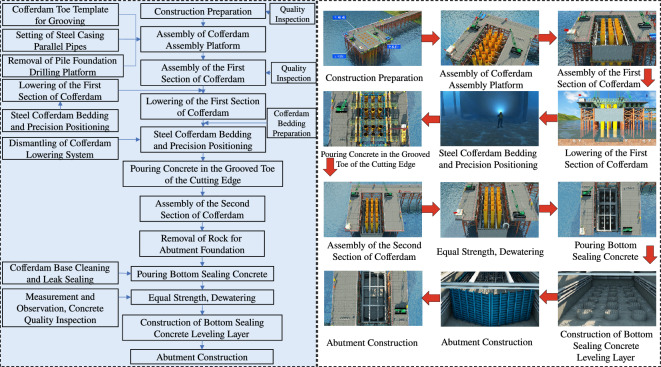
Construction simulation of pier 5 abutment of Chenxi Yuanshui extra-large bridge.

This key construction organization simulation encompasses:

- *This key construction organization simulation encompasses* Establishing a high-precision BIM model, continually optimizing the scaffold erection scheme to reduce material and labor consumption. Visual simulation standardizes the operation of lifting equipment during template installation and strictly adheres to relevant safety operating procedures to prevent accidents (Figure 5).
- *Specialized construction process simulation of tunnel construction* Utilizing the BIM model to simulate steps such as portal construction, advance support construction in the tunnel, advance long-pipe shed support in the open tunnel, excavation of the tunnel, lining of the tunnel, anti-drainage construction, composite waterproof layer construction, arch construction, lining construction, and secondary lining top-backfill grouting construction to control the quality and progress of the tunnel (Figure 6).
- *Simulation of widening construction plan for Chenxi South Hub Interchange Bridge* Developing a construction plan for a hub interchange involving traffic conversion, considering the impact on the existing highway to avoid traffic congestion and safety risks. Through integrated simulation of construction and traffic organizations, optimizing the construction plan, and reasonably arranging traffic control and diversion, traffic flow and safety are ensured (Figure 7).
- *Simulation of construction plan for Chenxi Yuanshui Extra-Large Bridge* Formulating a construction plan for an extra-large bridge spanning the Yuanshui waterway, innovatively using the “unsealed double-wall steel cofferdam method” The steel cofferdam is designed and manufactured modularly, assembled in sections, and constructed using the “track crane module assembly + jack lowering” method, increasing the difficulty of on-site construction organization and cofferdam lowering precision control. The simulation animation clearly displays the construction challenges of this project, reducing communication difficulties and improving owner satisfaction (Figure 8).

#### Traffic organization simulation for construction sections

Simulation of traffic organization in construction sections is crucial for evaluating control measures, comparing their effectiveness, and determining optimal traffic operation. This aids in optimizing traffic flow, ensuring a seamless and safe environment during construction.

**Figure 9:**
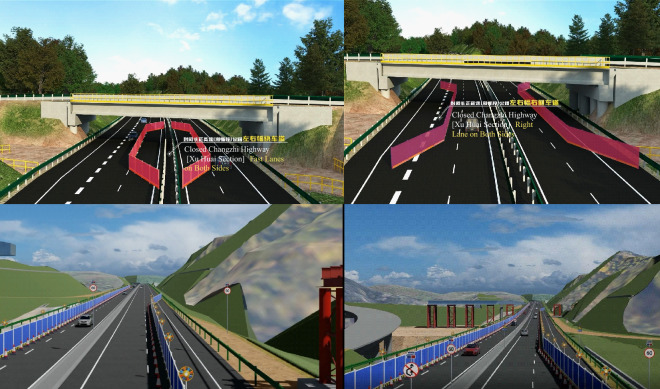
Traffic organization simulation.

In this project, anticipating potential traffic disruptions, emergency diversion plans were devised for the Yishu River Interchange (Section "Introduction") and Chenci South Hub Interchange (Section "Application of BIM Technology"). Traffic diversions and detour routes were assessed visually through simulations based on traffic volume and road conditions, incorporating over 20 types of temporary facilities using BIM models. Four primary categories of temporary signs and more than 10 structural components were strategically arranged for safety and warning purposes. (Figure 9).

Using the completed BIM model for dynamic display and visibility analysis, potential impacts of traffic organization schemes were predicted. Evaluating factors such as efficiency, safety, cost-effectiveness, sustainability, and stakeholder satisfaction led to adjustments in the scheme. This meticulous process ensured the selection of the optimal traffic organization, guaranteeing safety and unhindered vehicle flow during construction and minimizing impact on highway users.

#### 4D construction simulation

**Figure 10:**
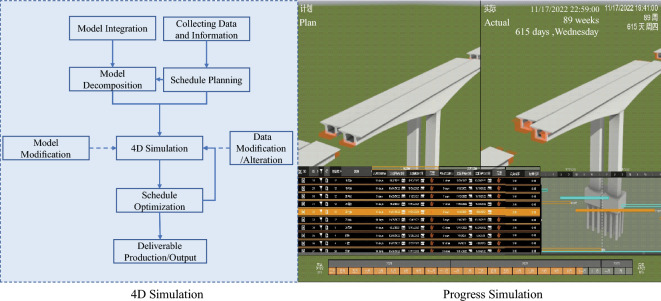
4D construction simulation.

4D construction simulation, also referred to as schedule simulation, incorporates time and action factors into components within the BIM model, illustrating the model’s evolution dynamically in 3D (Figure 10).

The primary tool employed for this schedule simulation was Fuzor software, in conjunction with the BIM model. After establishing the foundational model and preliminary schedule plan, scenario simulation was executed to render the entire project schedule visible and more comprehensible through an intuitive display and scenario optimization.

4D construction simulation operates as a real-time monitoring and management tool throughout the entirety of the project duration. By comparing planned construction with actual progress, construction simulation offers timely feedback, enabling swift identification of potential issues or delays. Adjustments can be promptly implemented by modifying the model or adjusting the schedule. This real-time, dynamic feedback mechanism robustly supports project management, ensuring that project progress aligns with the expected plan and mitigating potential risks. Through construction simulation, more efficient and precise engineering management can be attained throughout the entire project cycle.

#### Construction site layout simulation

Simulation of construction site layout replaces conventional 2D drawings with an intuitive three-dimensional model, integrated with a 3D environment, to more precisely anticipate and optimize the entire construction preparation process.

**Figure 11:**
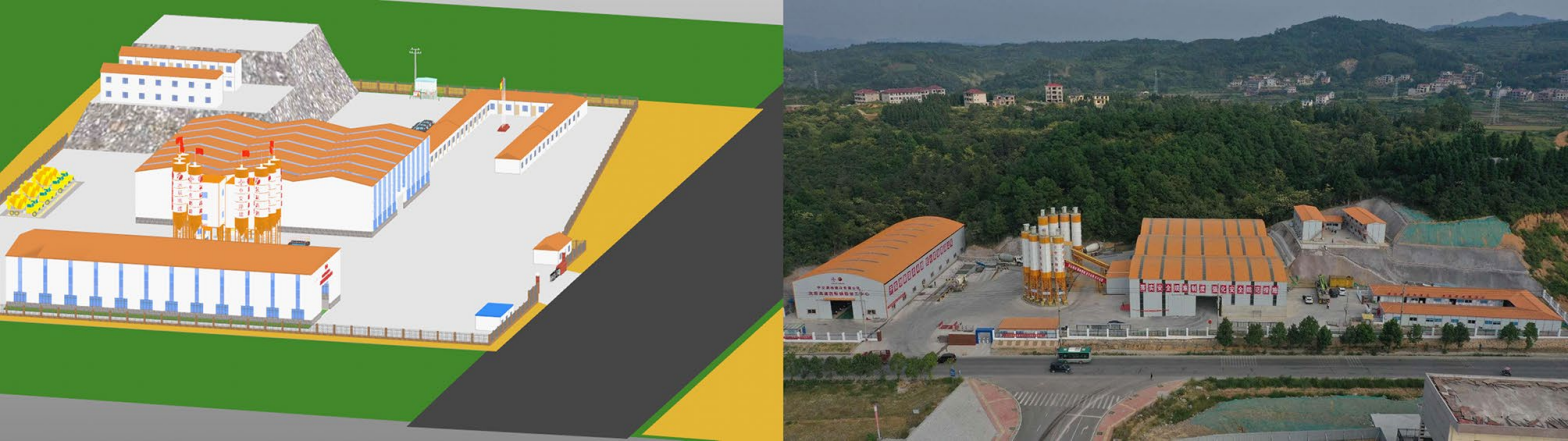
Mixing station at bid package No.4 of Yuanchen Highway.

This project integrates 4D simulation with modeling, incorporating machinery and workers to simulate site layout and logistics. Techniques such as construction activity conflict detection, resource allocation, and logical progress determine an optimal site layout plan, enhancing feasibility and streamlining task organization for increased effectiveness and cost-efficiency. The approach systematically considers construction factors, serving as a comprehensive guide for successful project implementation (Figure 11).

#### VR visualization disclosure

**Figure 12:**
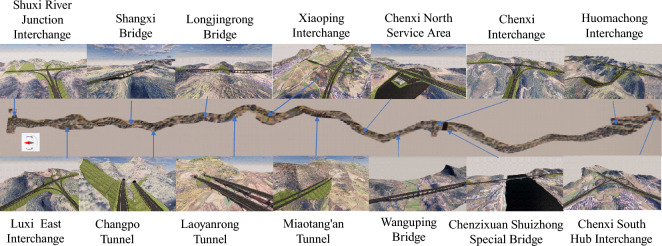
VR visualization disclosure.

Virtual Reality (VR) visualization integrates BIM models with VR experience devices, providing a dynamic exploration experience. This immersive approach enhances learning and engagement, addressing challenges like ambiguous drawings and incomplete communication. VR visualization surpasses traditional BIM model disclosures, offering an authentic encounter with the construction environment. It proactively reveals safety hazards and construction challenges, promoting safety awareness and ensuring construction quality (Figure 12).

#### Smart construction site applications

Modern construction sites leverage advanced information technologies, such as the IoT, cloud computing, big data, and artificial intelligence, for comprehensive monitoring and management of equipment, personnel, and materials. This integration enhances construction efficiency, ensures high-quality outcomes, and concurrently mitigates costs and risks. The applications of smart construction sites encompass various domains:

- Vehicle management:
  - Real-time collection of vehicle location and operational status data.
  - Continuous monitoring of vehicle fuel usage, triggering alarms for abrupt fuel decreases.
  - Detection of parameters like the rotation direction and speed of rotating equipment, facilitating the identification of reversing cement tankers and oil tankers.
  - Collection of front-end sensor data, transmitted to the cloud platform via IoT technology.
- Safety and environmental monitoring:
  - Monitoring the proper use of safety helmets, with automated reminders through broadcasting.
  - Establishment of defenses in critical hazard areas to prevent unauthorized access.
  - Implementation of a dust and noise detection system to ensure a conducive living environment for construction personnel.
  - Adoption of a personnel management system with facial recognition, intelligent sign-in, and electronic archiving of meeting images.

## The innovative application of BIM technology in the full-line construction of the project

Road infrastructure construction, more complex than building construction, presents challenges with expansive road networks covering many kilometers, potentially disrupting the surrounding environment. The Yan Chen highway project addresses these challenges, implementing innovative measures to enhance construction efficiency, quality, and cost-effectiveness. BIM technology is pivotal for the comprehensive management of the Yan Chen Highway, with key innovations including the following initiatives.

### BIM+GIS data integration technology for highway engineering

BIM+GIS technology merges BIM with GIS for efficient highway engineering information management. This integration minimizes spatial information costs by seamlessly connecting engineering data with the surrounding geographical environment. The technology facilitates comprehensive and intelligent support throughout the entire highway engineering process through continuous interaction between engineering components and the geographical context.

**Figure 13:**
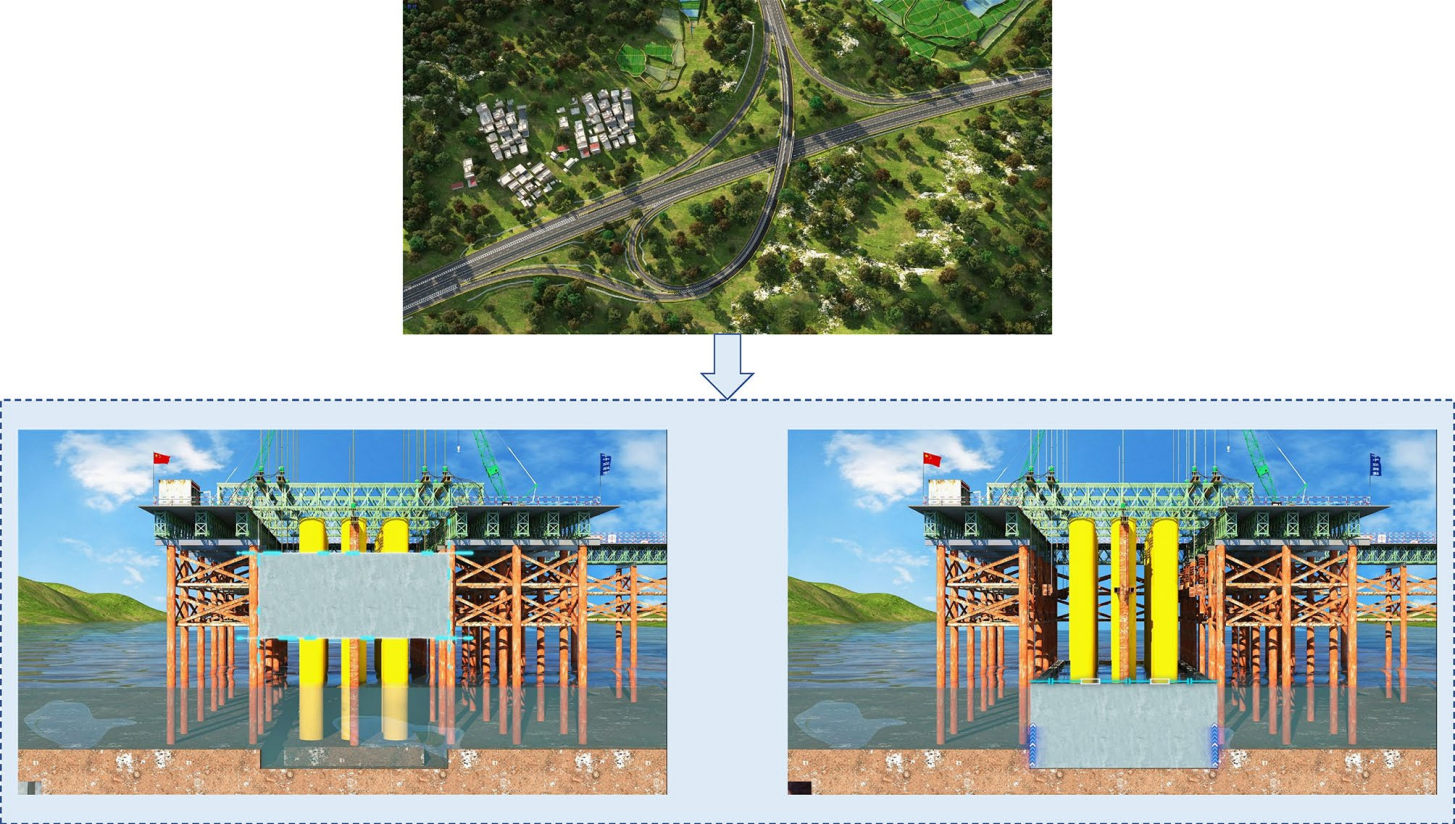
BIM+GIS technology guiding construction.

The implementation of Highways Engineering BIM+GIS Data Integration Technology involved a systematic series of steps, employing specialized software and equipment such as Bentley software, GIS software, drones, and LiDAR technology(Figure 13):

- *BIM model establishment *A detailed BIM model was meticulously crafted through BIM technology, seamlessly integrating high-precision spatial data encompassing images, point clouds, and 3D models collected via drones and LiDAR. This unified dataset, sharing a common coordinate system, facilitated seamless alignment and manipulation, presenting the highway project in a natural and cohesive manner.
- *Real-time monitoring with sensors* Real-time data from the physical system was transmitted to the BIM+GIS platform through sensors and monitoring devices. This facilitated continuous real-time monitoring and control of the real-world environment, offering dynamic insights into ongoing processes.
- *GIS spatial analysis* Leveraging the analytical capabilities of the GIS platform, spatial analysis, measurement, and simulation were employed to assess and optimize construction progress, operational statuses, and decision-making support.

### Construction of a BIM-based project command platform

This hybrid architecture, integrating Browser Side (web), Client Side (desktop), and Mobile End (mobile) through BIM+GIS technology, streamlines highway engineering data management. The platform, rooted in BIM technology, unifies information across project stages and establishes a digital twin model for enhanced project delivery and operation.

**Figure 14:**
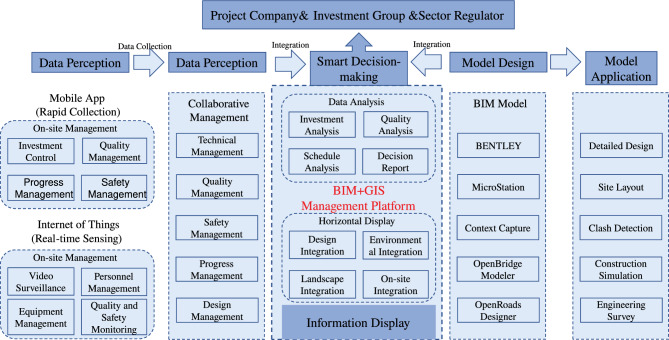
Project command platform construction process.

Through a BIM+GIS-based Management Cloud Platform, linked with a BIM coding system, it correlates BIM models with key modules like inspection, valuation, changes, progress reporting, and cost control. Mobile apps and IoT facilitate seamless data collection, providing real-time insights for effective on-site management. For specific construction processes, refer to (Figure 14). The platform enhances collaboration, ensuring visual, traceable, and sustainable attributes to project deliverables.

- *Technical management* Involves setting up a module for technical documents, integrating them with BIM components, and managing core technical documents like the startup report, construction organization design, and special construction plans. The platform supports online approval and viewing of technical documents at the model level, enabling users to access technical information based on the model’s location.
- *Progress management* Leverages BIM technology for information-based progress management, transforming the traditional approach to progress management. The system includes modules for planning and progress management, allowing simulation and analysis of the overall plan, annual plan, and current actual progress. This assists in analyzing delayed factors and critical points.

**Figure 15:**
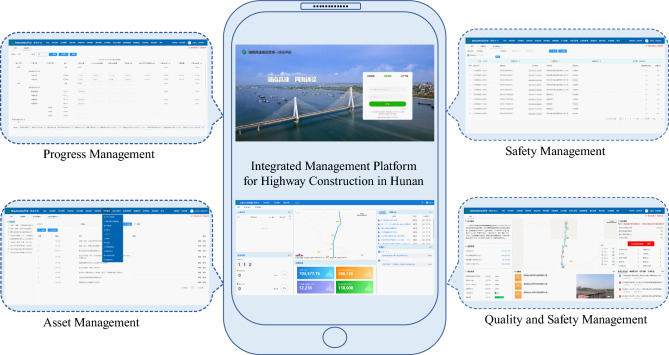
Hunan highway construction management integration platform.

This platform has the following functions and features:

- *Data integration and display* Integrates BIM and GIS data and functionalities, optimizing project construction by establishing a detailed BIM model during the construction phase. The platform provides comprehensive visual control, enhancing management capability for long-term and large-scale regional projects.
- *Business management and analysis* Integrates various business modules such as safety, quality, design, progress, technology, cost, and monitoring control. The platform utilizes cloud computing, artificial intelligence, and the IoT technologies to achieve automation, intelligence, and optimization of project management. It facilitates digital control and analysis of project aspects such as progress, cost, quality, and safety (Figure 15).
- *Data collection and sharing* Utilizes mobile apps and IoT devices for data collection, integrating processed data within the system. This assists all participating units in understanding on-site movements, facilitating data and information exchange among project participants.

In summary, through BIM+GIS data integration, this technology addresses real-world construction issues, optimizes construction, and reduces rework and material waste. It serves as the foundation for modern, intelligent, and centralized construction management. The platform significantly improves management efficiency, enhances the effectiveness of engineering management, achieves information interconnection, and promotes collaboration and dynamic management across multiple lines and departments.

## Discussion and impact

### Evaluation

In this endeavor, we conducted an exhaustive analysis encompassing social benefits, economic gains, and social evaluation.

1. *Social benefits* The project adopts a hybrid architecture (web, desktop, mobile) for integrated and visually collaborative management of highway construction using IoT, intelligent sensing, BIM, and GIS. This innovative approach enhances quality management by introducing pioneering methods and laying the foundation for IoT-based digital construction management, ultimately elevating the quality of highway construction throughout its lifecycle.
2. *Economic benefits *This project has brought multiple economic benefits. By establishing a BIM management platform, real-time dynamic management of project conditions was achieved, allowing for timely handling and reduction of construction risks. Additionally, through the efficient allocation of project materials, machinery, and personnel, utilization and efficiency were improved, reducing idle time and energy consumption, thus saving human and material resources. The project’s budgeted cost was 7.036 billion RMB (approximately 970 million USD). Despite significant macroeconomic fluctuations, not only was the risk of overspending avoided, but actual savings amounted to about 418 million RMB (approximately 57.68 million USD), achieving a cost-saving ratio of 5.94%. This included a 7.87% reduction in earthwork for subgrade, a 7.68% reduction in labor costs, and a 9.57% reduction in machinery rental fees. By enhancing communication efficiency through the management platform and optimizing construction plans, the construction period was successfully shortened. The original planned construction time was 36 months, but excluding force majeure, the actual completion time was 33 months, resulting in an efficiency improvement of approximately 8.3%. Several optimizations were made to the construction plans, particularly in solving the challenge of constructing the steel cofferdam for the No. 5 pier foundation of the Yuanshui Grand Bridge. BIM technology was applied across all four bid packages of the project, training nearly 30 engineers proficient in BIM technology, thus providing more talent resources for the industry.For detailed information, please refer to Table 1.
3. *Social evaluation* This project garnered multiple prestigious awards, underscoring its outstanding achievements:
  MIIT “Youlu Cup” National BIM Technology Competition Gold Award
  GUANGHONGAO Greater Bay Area Urban Architecture Society “Zhijian Cup” Silver Award
  China Technology Entrepreneurship Association “Gongchuang Cup” Intelligent Construction Technology Competition Third Prize
  These accolades signify our exceptional performance in technological innovation and project management, significantly enhancing the social evaluation of the project.

**Table 1:** Comparison of BIM and Non-BIM usage.

BIM usage	Earthwork for subgrade (10,000 m$^{3}$)	Material cost (billion CNY)	Labor cost (billion CNY)	Machinery rental cost (billion CNY)	Total cost (billion CNY)	Duration (months)
BIM	2163.89	4.28	0.86	1.48	6.62	33
Non-BIM	2348.79	4.47	0.93	1.64	7.04	36
Comparison	7.87%	4.25%	7.68%	9.57%	5.94%	8.3%

### Long-term impact

The enduring influence of BIM technology on the Yuanchen project encompasses a spectrum of dimensions. Not only does it enhance the efficiency and quality of the project, but it also facilitates the digital transformation and sustainable development of road infrastructure engineering. Key facets encompass:

- *Enhancement in construction management* This involves refining engineering processes, minimizing waste and risks, and amplifying project quality and safety through the adept utilization of construction simulation and schedule control.
- *Augmented efficiency in operation and maintenance* This aspect aids personnel in optimizing equipment maintenance and highway management, consequently mitigating equipment failure rates and reducing operational costs.
- *Heightened sustainability* Enhancing project sustainability involves several measures such as analyzing and simulating energy efficiency and material selection, streamlining construction processes, reducing the number of bridge piers, and implementing meticulous water resource protection strategies. These actions aim to achieve lower energy consumption and emissions, thereby improving the environmental friendliness of the project.

### Limitations

While BIM technology played a pivotal role in facilitating seamless information transfer, sharing, and integration across various construction stages in this project, limitations are discernible in two key areas:

- *Realize 5D cost model and 6D safety model* Despite notable advancements in aspects such as quantity surveying and schedule simulation, the attainment of 5D cost models and 6D safety models lags. The absence of intelligent management in costs and safety may result in less precise and comprehensive project oversight. Future endeavors should prioritize overcoming this constraint to achieve a more thorough and intelligent approach to project management.
- *AI integration* The project did not align with the trajectory of Artificial Intelligence (AI). The integration of BIM with AI has the potential to expedite model generation, enable intelligent recognition, and automate optimization of BIM models. This could significantly diminish modeling time, enhance modeling quality, and elevate the overall intelligence of BIM technology.

## Conclusion

This paper conducts a case study on the Yuanchen Highway project, emphasizing the application and impact of BIM technology in road engineering. The objective is to showcase the value of BIM technology in the road infrastructure sector, offering novel insights and directions for its further development.

This paper first analyzes the current status and existing issues of BIM technology application in the field of road infrastructure, pointing out that there is still significant development space and potential in this area, and that more innovation and transformation are urgently needed. This project successfully implemented research findings by integrating several cutting-edge technologies, including BIM, GIS, IoT, and VR. Utilizing BIM technology, high-precision 3D models were created, and during the construction process, detailed site analysis was conducted in conjunction with GIS data. The deployed IoT sensor network provided real-time monitoring of environmental conditions and progress data on the construction site, ensuring the safety and efficiency of the construction process. Meanwhile, the application of VR technology provided an immersive virtual simulation environment, assisting in design reviews and construction simulations, greatly enhancing communication efficiency among project parties and the feasibility of plans.

The paper then focuses on the technical and managerial innovations of the project command platform based on BIM+GIS technology in the Yuancheng Expressway project. Through case studies, it demonstrates the feasibility and effectiveness of BIM technology in road engineering, as well as its advantages in improving project quality, efficiency, and energy savings. Finally, the paper summarizes the experiences and lessons learned from the application of BIM technology in road engineering, and proposes recommendations and prospects for the development of BIM technology in the field of road infrastructure.

The research not only enhances the precision and efficiency of project management but also provides valuable reference significance for promoting the application and development of BIM technology in the field of road infrastructure. It also offers a replicable BIM technology application model and case study for other similar engineering projects. This research offers valuable references and insights for promoting the application and development of BIM technology in the road infrastructure sector. It presents a replicable BIM technology application model and case for similar engineering projects. However, the study acknowledges limitations, such as the need to enhance the depth and breadth of BIM technology application, explore integration and innovation with other information technologies, and improve standardization. The hope is that this research inspires more researchers and practitioners to focus on and participate in the application and development of BIM technology in this field, contributing to the establishment of intelligent transportation and a better society.

## Data Availability

The datasets used during the current study available from the corresponding author on reasonable request.
